# A Novel Combination of Probiotic Supplements Reduces Gut Permeability, Oxidative Stress, and Inflammation in Undernourished Adults: A Randomized Controlled Trial

**DOI:** 10.1002/fsn3.71915

**Published:** 2026-05-27

**Authors:** Maryam Ahmadi‐Khorram, Alireza Hatami, Mohammad Hadi Eskandari, Asma Afshari, Ali Jafarzadeh Esfehani, Parastoo Asghari, Elyas Nattagh‐Eshtivani, Mohsen Nematy

**Affiliations:** ^1^ Department of Nutrition, Faculty of Medicine Mashhad University of Medical Sciences Mashhad Iran; ^2^ Student Research Committee Mashhad University of Medical Sciences Mashhad Iran; ^3^ Department of Food Science and Technology Shiraz University Shiraz Iran; ^4^ Metabolic Syndrome Research Center Mashhad University of Medical Sciences Mashhad Iran; ^5^ Department of Nutrition, Food Sciences and Clinical Biochemistry, School of Medicine, Social Determinants of Health Research Center Gonabad University of Medical Science Gonabad Iran

**Keywords:** gut barrier function, *Lactobacillus*, oxidative stress, probiotics, undernutrition, zonulin

## Abstract

Undernutrition is associated with intestinal barrier dysfunction, oxidative stress, and systemic inflammation. This randomized controlled trial investigated the effects of an 8‐week multi‐strain probiotic supplementation combined with an energy‐surplus diet in underweight adults. In this double‐blind, placebo‐controlled trial (IRCT20230310057667N1), 100 underweight adults (BMI < 18.5 kg/m^2^, aged 18–60 years) were randomized (1:1) using sex‐stratified block randomization (block size 4) to receive either two daily probiotic capsules ( *Lactobacillus acidophilus* LA5 10^9^ CFU, *Lactobacillus rhamnosus* GG 10^9^ CFU, *Lactobacillus casei* 10^9^ CFU) plus a 500 kcal/day energy‐surplus diet, or identical placebo plus the same diet. Primary outcome was change in serum zonulin concentration. Secondary outcomes included markers of oxidative stress and inflammation. Per‐protocol analysis was conducted on 95 participants who completed the study. Serum zonulin concentration decreased by a mean of 0.98 ± 1.61 ng/mL in the probiotic group versus 0.22 ± 1.12 ng/mL in the placebo group (*p* = 0.047). Median changes in secondary outcomes (probiotic versus placebo) were as follows: total antioxidant capacity +0.40 versus −0.01 mmol/L, total oxidant status −4.22 versus +0.79 μmol/L, glutathione peroxidase +4.0 versus −0.8 nmol/mL, malondialdehyde −0.046 versus +0.006 nmol/mL, C‐reactive protein −0.50 versus +0.10 mg/L, and 1‐h erythrocyte sedimentation rate −1 versus +1 mm/h. The between‐group differences were statistically significant for all outcomes (*p* < 0.001 for secondary outcomes; *p* = 0.047 for serum zonulin). Multi‐strain probiotic supplementation combined with a weight‐gain diet significantly improves gut barrier integrity, reduces oxidative stress and attenuates systemic inflammation in undernourished adults, offering a promising adjunctive nutritional intervention.

## Graphical abstract

Multi‐strain probiotics + energy‐surplus diet improve gut barrier, reduce oxidative stress & inflammation in undernourished adults—8‐week RCT.

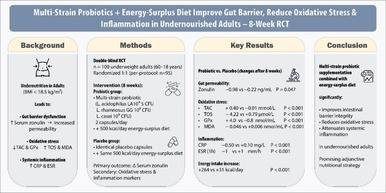

**Abbreviations**
BMI: body mass index
CBC: complete blood count
CFU: colony‐forming unit
CRP: C‐reactive protein
ESR: erythrocyte sedimentation rate
GPx: glutathione peroxidase
HC: hip circumference
MDA: malondialdehyde
MLR: monocyte‐to‐lymphocyte ratio
NLR: neutrophil‐to‐lymphocyte ratio
RDW‐SD: red cell distribution width—standard deviation
TAC: total antioxidant capacity
TOS: total oxidant status
WC: waist circumference

## 1 Introduction

Undernutrition, characterized by insufficient energy and nutrient intake, affects approximately 390 million adults globally, leading to underweight status, wasting, and increased morbidity (Hegazi et al. 2024; World Health Organization 2024). Undernutrition disrupts gut microbiota, compromises intestinal barrier function, and promotes systemic inflammation and oxidative stress, which impair nutrient absorption and exacerbate health complications (Genton et al. 2015; Purnasari et al. 2021; Thompson et al. 2021). Dysbiosis due to the imbalance in the gut microbiome compromises intestinal permeability by altering tight junction proteins, including zonulin and occludin (Christian et al. 2020; Levy et al. 2017; Pino et al. 2021; Stärkel et al. 2018). Altered tight junctions allow endotoxins to enter systemic circulation and exacerbate inflammation and undernutrition‐related complications (Mostafavi Abdolmaleky and Zhou 2024; Van Krimpen et al. 2021).

Probiotics are live microorganisms conferring health benefits when consumed in adequate amounts (Hotel 2001). Probiotics recolonization restores gut microbiota balance and enhances intestinal barrier function (Gou et al. 2022). Research demonstrates that *Lactobacillus* species reduce inflammation and oxidative stress by producing short‐chain fatty acids (SCFAs) and boosting antioxidant enzyme activity (e.g., glutathione peroxidase and superoxide dismutase) (Chaiyasut et al. 2022; Fluitman et al. 2017; Zolghadrpour et al. 2024). Specifically, *Lactobacillus acidophilus* has been shown to improve gut permeability, *Lactobacillus casei* supports mucosal immunity and reduce inflammation, and *Lactobacillus rhamnosus* was found to promote weight gain in malnourished populations (Gauffin et al. 2002; Million et al. 2012; Pan et al. 2021; Vendt et al. 2006). However, evidence on the efficacy of specific probiotic strains in enhancing gut barrier function are conflicting, with some studies reporting moderate improvements in tight junction integrity and others depicting minimal impact, due to strain‐specific effects or differences in study design (Al‐Sadi et al. 2021). Furthermore, while certain Lactobacillus strains promote weight gain, others may induce weight loss, necessitating strain‐specific interventions (Million et al. 2012).

The current body of probiotic research is especially focused on overweight populations or populations with diseases. However, the role of probiotics administration in underweight adults, particularly in combination with weight‐gain dietary interventions, remains underexplored (Kadooka et al. 2013; Kazemi et al. 2020; Pan et al. 2021; Sanchez et al. 2014). This study investigates the synergistic effects of an 8‐week course of multi‐strain probiotic supplement ( *Lactobacillus acidophilus* , *Lactobacillus casei* , and *Lactobacillus rhamnosus* ) on antioxidant defense and zonulin levels (a marker of gut permeability) in combination with a tailored dietary intervention in underweight adults. By assessing zonulin, inflammation, and oxidative stress, this study aims to develop targeted nutritional interventions for undernutrition and advance clinical nutrition practices.

## 2 Methods and Materials

### 2.1 Study Design

The present study was a double‐blind, randomized, placebo‐controlled trial designed to evaluate the effects of an 8‐week intervention combining a probiotic supplement (containing *Lactobacillus rhamnosus GG*, *Lactobacillus acidophilus* , and *Lactobacillus casei* ) with a weight gain diet on gut barrier function, oxidative stress markers, and inflammatory responses in underweight adults. The present study was conducted at the Nutrition Specialty Clinic of the Imam Reza Hospital in Mashhad, Iran, from August 2024 to February 2025. The study complied with the ethical principles of the Declaration of Helsinki. Ethical clearance was granted by the Mashhad University of Medical Sciences Ethics Committee (codes: IR.MUMS.MEDICAL.REC.1401.589, IR.MUMS.MEDICAL.REC.1402.057), and the trial was registered with the Iranian Registry of Clinical Trials (IRCT20230310057667N1) (Issue date 30/03/2023), https://irct.behdasht.gov.ir/trial/69130. This study was financially supported by grants from Mashhad University of Medical Sciences (MUMS), Mashhad, Iran (award/grant number: 4012306).

### 2.2 Participants

Eligible participants were outpatients aged 18–60 years with undernutrition defined as body mass index (BMI) below 18.5 kg/m^2^, who did not have underlying medical conditions, were able to read and write, and were willing to participate in the study by the provision of written informed consent. Exclusion criteria encompassed pregnancy or lactation, chronic conditions (e.g., diabetes, cancer, liver or kidney disease, inflammatory bowel disease, or irritable bowel syndrome), use of medications influencing appetite or weight, consumption of antibiotics or probiotic‐containing products within the past 3 months, alcohol use, or regular intake of probiotic‐rich foods.

Participants were withdrawn from the study if they chose to discontinue participation in the study, became pregnant, developed adverse reactions to the probiotic or placebo, or non‐adherence defined as less than 80% adherence to the intervention protocol.

The sample size was determined based on prior research by Wilms et al. examining zonulin changes in healthy individuals (Wilms et al. 2016). Calculation determined 42 participants per group. Accounting for an 18% dropout rate, 50 individuals were recruited per group, totaling 100 participants.

### 2.3 Randomization and Blinding

A stratified randomization approach was employed to ensure balanced group allocation by sex. An independent statistician (not otherwise involved in the trial) generated the random allocation sequence using a web‐based tool (https://www.sealedenvelope.com), producing 25 blocks of size 4, stratified by sex.

Eligible participants were enrolled by the principal investigator and a research coordinator after providing written informed consent. Allocation to the intervention or placebo group was implemented by a blinded study pharmacist, who assigned participants according to the pre‐generated sequence using sequentially numbered, sealed, opaque envelopes. Allocation concealment was maintained until after enrollment, and the sequence remained concealed from participants, enrolling staff, intervention administrators, outcome assessors, and data analysts throughout the trial.

### 2.4 Instruments

At baseline, participants completed a detailed questionnaire including demographic details (e.g., age, occupation, education), socio‐economic status (e.g., household size, housing conditions), medical history, and current medication or supplement use.

### 2.5 Biochemical Evaluations

Blood samples were obtained at the baseline and the end of the 8th week following an overnight fast of 12 h. Whole blood was centrifuged at 3500 rpm for 10 min to isolate serum. Analyses were performed at a private laboratory in Mashhad, Iran. Serum C‐reactive protein (CRP), erythrocyte sedimentation rate (ESR) at 1 and 2 h, and complete blood count (CBC) were measured immediately following blood collection. Remaining serum aliquots were frozen at −70°C for subsequent assays. To assess gut barrier integrity, plasma levels of active, uncleaved zonulin were quantified using a Zonulin ELISA Kit (Biorbyt, UK). Malondialdehyde (MDA), an indicator of oxidative stress, was measured in serum via a spectrophotometric assay based on its reaction with thiobarbituric acid (TBA). Additional oxidative stress markers, including total antioxidant capacity (TAC), total oxidant status (TOS), and glutathione peroxidase (GPx), were evaluated using commercial kits (Navand Salamat Co., Urmia, Iran) and spectrophotometric techniques. Hematological‐inflammatory indices, specifically the neutrophil‐to‐lymphocyte ratio (NLR) and monocyte‐to‐lymphocyte ratio (MLR), were derived by dividing neutrophil or platelet counts by lymphocyte counts, respectively (Diem et al. 2017).

The energy requirements for each individual were calculated via the Mifflin equation (Mifflin et al. 1990). All participants received individualized dietary counseling to achieve an additional energy surplus of approximately 500 kcal/day above estimated requirements, with a recommended overall macronutrient distribution of 55% carbohydrates, 30% fats, and 15% proteins to support healthy weight gain. Participants were instructed to preserve the structure and food types of their habitual diet as much as possible while incorporating additional calorie‐dense foods/snacks to meet the energy surplus target. They were also advised to avoid consuming additional probiotic or synbiotic products during the study. Dietary intake and physical activity were assessed at baseline and the end of the study. Physical activity levels were measured using the validated Persian translation of the International Physical Activity Questionnaire (IPAQ) (Vasheghani‐Farahani et al. 2011). Based on weekly activity duration, participants were classified as having mild (< 600 min), moderate (600–3000 min), or high (> 3000 min) activity levels.

### 2.6 Intervention

Participants were assigned to either the intervention or control group and instructed to take two capsules daily—probiotic or placebo—for 8 weeks. Probiotic capsules, manufactured by *Pardis Roshd* Company (Iran), contained *Lactobacillus acidophilus* LA5 4356 (10^9^ CFU/capsule), *Lactobacillus rhamnosus* GG53103 (10^9^ CFU/capsule), *Lactobacillus casei* (10^9^ CFU/capsule), and a maltodextrin filler. Placebo capsules, also produced by *Pardis Roshd* Company, contained only maltodextrin and were designed to match the probiotic capsules in appearance, size, taste, and odor.

### 2.7 Statistical Analysis

The primary endpoint was the difference in gut permeability, assessed by serum zonulin concentration, between the two groups. Secondary endpoints include oxidative stress (measured by serum malondialdehyde [MDA], total antioxidant capacity [TAC], total oxidant status [TOS], and glutathione peroxidase [GPx]), inflammation (assessed via C‐reactive protein [CRP] and erythrocyte sedimentation rate [ESR]). Statistical analyses were performed using SPSS software (version 26). The per‐protocol approach was applied for all analyses. Normality distribution of continuous data was assessed using the Kolmogorov–Smirnov test. Continuous data were reported as means ± standard deviations for normally distributed data and median and interquartile range (IQR) for non‐normally distributed data. Comparison of continuous data between groups was performed using independent *t*‐tests for normally distributed data and Mann–Whitney *U* tests for non‐normally distributed data. Within‐group comparisons were conducted using paired *t*‐tests for normally distributed data and Wilcoxon signed‐rank tests for non‐normally distributed data. Chi‐square tests were applied to compare the distribution of categorical data between groups.

### 2.8 Adverse Events

No adverse events were reported during the 8‐week intervention in either the probiotic or placebo group. Gastrointestinal symptoms were actively monitored via weekly contact and follow‐up visits, but none occurred. No serious adverse events or withdrawals due to side effects were observed.

## 3 Results

**FIGURE 1:**
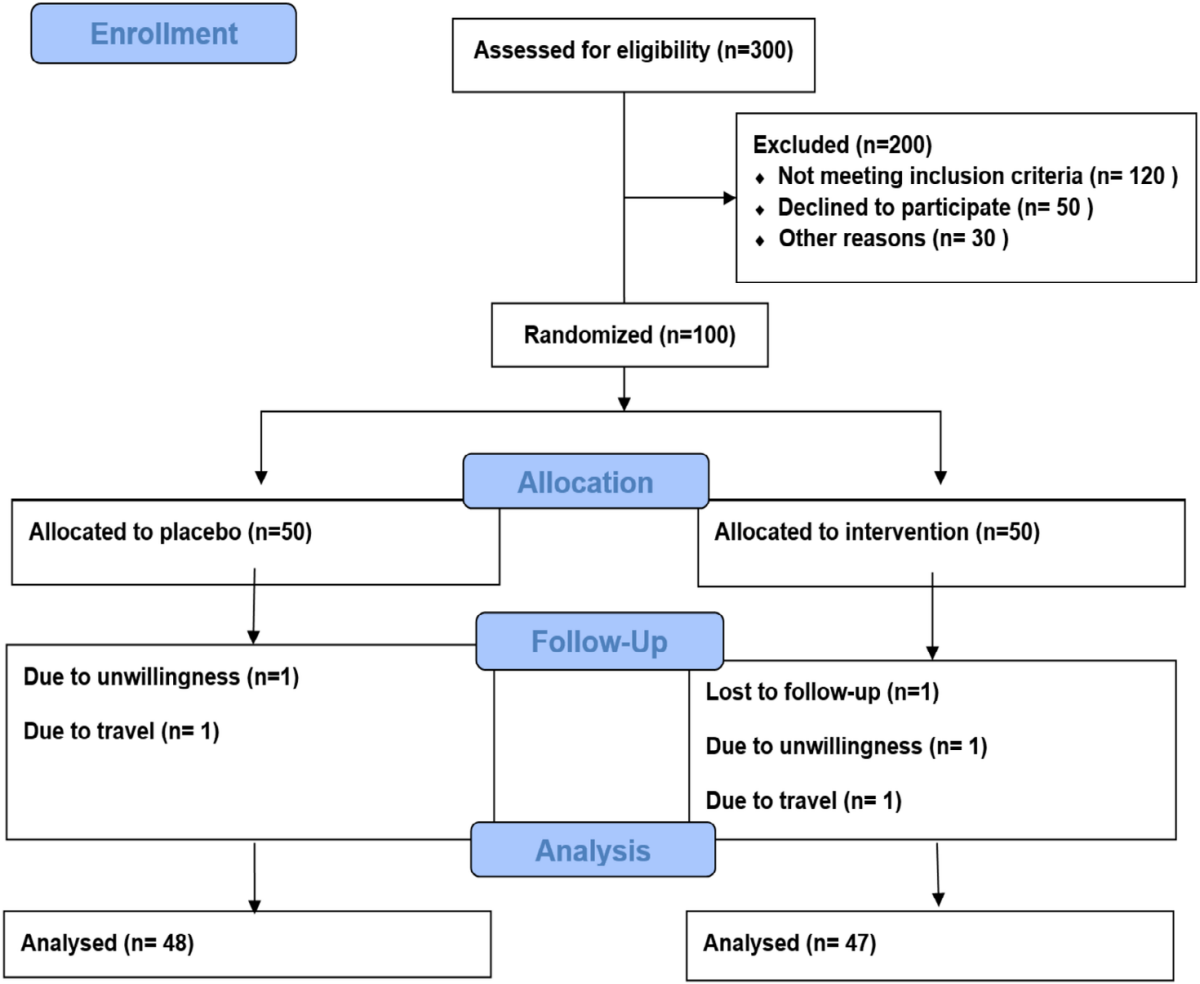
Flowchart of the study. Including patient screening, enrollment, and randomization. Dropouts occurred due to unwillingness and travel.

A total of 95 participants completed the 8‐week intervention, with 47 individuals in the probiotic group and 48 in the placebo group (Figure 1). At baseline, there were no statistically significant differences between the groups in terms of demographic, anthropometric, and dietary characteristics (all *p* > 0.05), confirming adequate matching of the participants. However, statistically significant baseline differences were observed in marital status and hip circumference, although these differences are unlikely to have substantially affected the primary and secondary outcomes (Table 1).

**TABLE 1:** Baseline demographic, anthropometric, and dietary characteristics of the study participants.

Characteristic	Placebo group (*n* = 48)	Probiotic group (*n* = 47)	*p*
Age (years)	25.9 ± 8.0	26.5 ± 7.1	0.723
Sex (male)	20 (41.7%)	25 (53.2%)	0.261*
Marital status (married)	3 (6.3%)	16 (34.0%)	0.001*
University education	41 (85.4%)	41 (87.2%)	0.797*
Physical activity level			0.360*
Mild	24 (50.0%)	27 (57.4%)	
Moderate	15 (31.3%)	16 (34.0%)	
Severe	9 (18.8%)	4 (8.5%)	
Energy intake (kcal/day)	1495 ± 336	1530 ± 335	0.630
Carbohydrate intake (g/day)	206 ± 46	210 ± 46	0.610
Protein intake (g/day)	67 ± 15	69 ± 15	0.620
Fat intake (g/day)	50 ± 11	51 ± 11	0.600
Dietary fiber (g/day)	20.9 ± 4.7	21.4 ± 4.7	0.610
Weight (kg)	48.4 ± 7.0	50.3 ± 8.0	0.216
BMI (kg/m^2^)	17.3 ± 1.3	17.4 ± 1.5	0.680
Waist circumference (cm)	71.3 ± 6.6	71.0 ± 4.8	0.820
Hip circumference (cm)	86.8 ± 6.9	89.2 ± 4.1	0.039

*Note:* Data are mean ± SD or *n* (%). *p*‐values from independent *t*‐test or *chi‐square test.

Compared with the placebo group, probiotic supplementation resulted in significantly greater improvements in all oxidative‐stress and inflammatory markers. Total antioxidant capacity increased, whereas total oxidant status, malondialdehyde, C‐reactive protein, and 1‐h erythrocyte sedimentation rate decreased markedly only in the probiotic group. Glutathione peroxidase activity increased significantly only in the probiotic group (*p* < 0.001) (Tables 2 and 3).

**TABLE 2:** Energy intake at baseline, week 8, and changes during the intervention.

Parameter	Placebo group (*n* = 48)	Probiotic group (*n* = 47)	Between‐group *p*‐value
Energy intake (kcal/day)			
Week 0 (baseline)	1495.14 ± 336	1529.79 ± 335	0.630
Week 8 (endline)	1525.89 ± 360.14	1793.34 ± 449.92	0.050
Within‐group change	+30.75 (−2.28, 63.78)	+263.55 (199.4, 327.7)	—
Within‐group *p*‐value	0.067	0.001	—
Between‐group difference in change (MD, 95% CI)	—	—	232.96 (163.06, 302.87) *p* < 0.001

*Note:* Data are presented as mean ± SD for absolute values and mean (95% CI) for changes. Between‐group comparisons at baseline and week 8 were performed using independent *t*‐test. Within‐group changes assessed using paired *t*‐test. Between‐group difference in change assessed using independent *t*‐test on the delta values.

**TABLE 3:** Oxidative stress and inflammatory markers at baseline and week 8.

Variable	Placebo (*n* = 48)	Probiotic (*n* = 47)	*p*‐value for change^a^
TAC (mmol/L)			
Baseline	0.67 (0.48–0.97)	0.57 (0.37–0.87)	< 0.001
Week 8	0.60 (0.38–0.98)	0.96 (0.69–1.39)	
Median change (IQR)	−0.008 (−0.10 to 0.50)	0.399 (0.20 to 0.61)	
Within‐group *p*‐value	0.060	< 0.001	
TOS (μmol/L)			
Baseline	3.90 (1.10–6.69)	7.47 (3.25–12.84)	< 0.001
Week 8	4.44 (2.79–8.02)	2.87 (0.41–6.19)	
Median change (IQR)	0.79 (−0.87 to 2.79)	−4.22 (−8.07 to −0.55)	
Within‐group *p*‐value	0.010	< 0.001	
GPx (nmol/mL)			
Baseline	29.56 (27.45–30.51)	25.81 (24.63–27.83)	< 0.001
Week 8	28.75 (26.67–29.23)	30.04 (26.86–34.98)	
Median change (IQR)	−0.78 (−2.58 to 0.00)	4.00 (1.88 to 6.58)	
Within‐group *p*‐value	0.001	< 0.001	
MDA (nmol/mL)			
Baseline	0.05 (0.02–0.09)	0.09 (0.07–0.12)	< 0.001
Week 8	0.10 (0.05–0.13)	0.04 (0.02–0.06)	
Median change (IQR)	0.006 (0.00 to 0.104)	−0.046 (−0.088 to −0.014)	
Within‐group *p*‐value	0.001	< 0.001	
CRP (mg/L)			
Baseline	1.75 (1.00–4.20)	1.00 (0.50–3.00)	< 0.001
Week 8	4.00 (1.92–4.80)	1.00 (0.30–1.20)	
Median change (IQR)	0.10 (−0.35 to 3.67)	−0.50 (−2.00 to 0.20)	
Within‐group *p*‐value	0.020	0.036	
ESR1 (mm/h)			
Baseline	3 (2–4.75)	5 (3–10)	< 0.001
Week 8	4 (3–6.75)	3 (3–6)	
Median change (IQR)	1 (0 to 2)	−1 (−4 to 0)	
Within‐group *p*‐value	0.001	< 0.001	

*Note:* Data are median (interquartile range).

^a^ Between‐group comparison of changes from baseline performed using the Mann–Whitney U test. Within‐group comparisons were performed using the Wilcoxon signed‐rank test.

**FIGURE 2:**
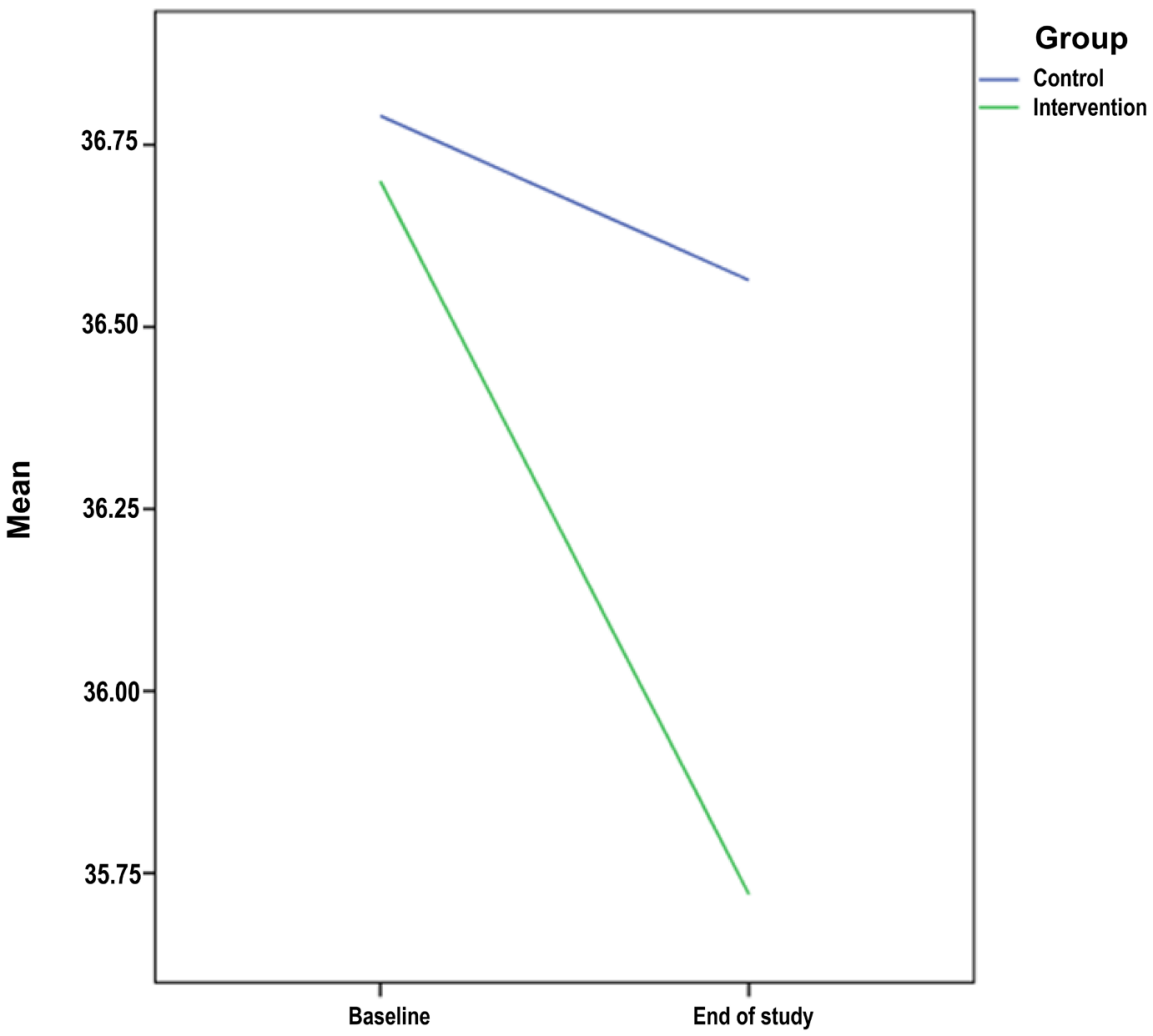
Changes in zonulin levels over the study period in the intervention and control groups.

Serum zonulin concentration (primary outcome) decreased from 36.70 (36.07, 37.32) to 35.72 (35.15, 36.28) ng/mL in the probiotic group (*p* < 0.001) but not in the placebo group (−0.22 (−0.65, 0.2) ng/mL, *p* = 0.290). The between‐group difference in change was statistically significant (*p* = 0.047; Table 4, Figure 2).

**TABLE 4:** Serum zonulin concentrations (primary outcome) at baseline and week 8.

Variable	Placebo group (*n* = 48)	Probiotic group (*n* = 47)	*p*
Zonulin (ng/mL), baseline	36.78 ± 1.98	36.70 ± 2.12	0.830
Zonulin (ng/mL), week 8	36.56 ± 1.95	35.72 ± 1.92	0.037
Change from baseline	−0.22 ± 1.12	−0.98 ± 1.61	0.047
Within‐group *p*‐value	0.290	< 0.001	—

*Note:* Data are presented as mean ± SD. Between‐group comparisons were performed using independent *t*‐test (baseline and week 8). Within‐group comparisons were performed using paired *t*‐test.

No significant effects of the intervention were observed on hematological inflammatory indices (neutrophil‐to‐lymphocyte ratio, monocyte‐to‐lymphocyte ratio, or RDW‐SD; Table 5). Erythrocyte sedimentation rate measured at 2 h showed identical trends to the 1‐h value and is therefore not reported.

**TABLE 5:** Hematological inflammatory indices at baseline and week 8.

Variable	Placebo group (*n* = 48)	Probiotic group (*n* = 47)	*p* ^a^ (change)
RDW‐SD (fL)			
Baseline	47.9 (46.5–49.4)	47.1 (45.6–48.7)	
Week 8	47.5 (43.9–48.5)	46.2 (45.1–47.4)	
Change	−0.9 (−3.5 to 0.7)	−0.6 (−1.8 to 0.0)	0.447
Within‐group *p*‐value	0.001	0.004	
Neutrophil‐to‐lymphocyte ratio (NLR)			
Baseline	1.75 (1.32–2.68)	1.86 (1.61–2.46)	
Week 8	1.64 (1.25–2.47)	2.04 (1.31–2.48)	
Change	−0.12 (−0.42 to 0.31)	0.03 (−0.43 to 0.47)	0.450
Within‐group *p*‐value	0.383	0.700	
Monocyte‐to‐lymphocyte ratio (MLR)			
Baseline	0.15 (0.11–0.22)	0.15 (0.13–0.19)	
Week 8	0.14 (0.11–0.18)	0.16 (0.13–0.22)	
Change	−0.01 (−0.04 to 0.03)	0.01 (−0.03 to 0.05)	0.130
Within‐group *p*‐value	0.300	0.280	

*Note:* Data are presented as median (interquartile range).

^a^ Between‐group comparison of changes was performed using the Mann–Whitney U test. Within‐group comparisons were performed using the Wilcoxon signed‐rank test. No significant between‐group differences were observed for any hematological inflammatory index (all *p* > 0.05).

## 4 Discussion

To the best of our knowledge, this randomized, double‐blind clinical trial was the first to evaluate the effects of an 8‐week multi‐strain probiotic intervention, comprising *Lactobacillus rhamnosus GG*, *Lactobacillus acidophilus* , and *Lactobacillus casei* , on gut permeability, oxidative stress, and inflammation in underweight adults. Our findings demonstrate significant improvements in serum zonulin, TAC, TOS, GPx, MDA, CRP, and ESR in the probiotic group compared to baseline. In contrast, the placebo group showed increased TOS and MDA and decreased GPx, with significant between‐group differences for most markers. These results suggest that multi‐strain probiotics may mitigate undernutrition‐related complications by enhancing gut barrier function, reducing oxidative damage, and attenuating systemic inflammation.

Undernutrition, characterized by inadequate nutrient intake, impairs pancreatic function, intestinal blood flow, villus structure, and gut barrier integrity, leading to compromised nutrient absorption and altered gut microbiota composition (Genton et al. 2015; Purnasari et al. 2021; Saunders and Smith 2010). Current therapeutic approaches for undernutrition often lack efficacy (Christian et al. 2020), highlighting the need for novel interventions like probiotics to complement dietary treatments (Kane et al. 2015).

### 4.1 Gut Permeability

The significant between‐group difference in serum zonulin reduction (−0.98 ± 1.61 ng/mL in the probiotic group vs. −0.22 ± 1.12 ng/mL in the placebo group; *p* = 0.047) indicates that multi‐strain probiotic supplementation provided additional improvement in intestinal barrier integrity beyond the energy‐surplus diet alone. Although modest (1 ng/mL), this reduction is physiologically relevant in undernourished adults, as even small decreases can reflect improved tight junction integrity and reduced permeability, thereby helping mitigate nutrient malabsorption and systemic complications. Baseline levels suggest mild barrier dysfunction, and the observed change aligns with prior probiotic studies linking zonulin reductions to lower endotoxemia and inflammation (Zheng et al. 2023).

This finding was particularly relevant for underweight adults, as malnutrition‐induced intestinal barrier dysfunction exacerbates nutrient malabsorption (Christian et al. 2020; Stärkel et al. 2018). Zonulin, a modulator of tight junction integrity, serves as an innovative and underexplored biomarker to assess undernutrition severity and treatment response. The findings of the present study aligned with previous studies reporting decreased zonulin levels after probiotic supplementation in stressed individuals and those with osteoarthritis (Karim et al. 2024; Mk et al. 2018). However, a trial in patients with type 2 diabetes found no effect on gut permeability after 12 weeks, possibly due to disease‐specific microbiota dysbiosis or longer intervention duration (Toejing et al. 2021). A recent meta‐analysis supports our observations, indicating that probiotics reduced serum zonulin levels, with efficacy influenced by intervention duration and population characteristics (Zheng et al. 2023). The mechanism for this effect can be due to the effects of probiotics in strengthening the intestinal barrier by upregulating tight junction proteins (e.g., ZO‐1, claudin‐1), promoting epithelial cell proliferation, and enhancing mucin production, particularly MUC2, which bolsters the protective mucus layer (Gou et al. 2022; La Fata et al. 2018). Specifically, *L. acidophilus* may reduce zonulin levels by restoring gut barrier integrity (Liu et al. 2015, 2013), while *L. casei* supports mucosal immunity (Gauffin et al. 2002). Additionally, probiotic‐derived short‐chain fatty acids (SCFAs), such as butyrate, provide energy to colonocytes, reinforce tight junction integrity, and exert anti‐inflammatory effects (Hodgkinson et al. 2023). These changes may enhance nutrient absorption, addressing a critical challenge in undernutrition (Hegazi et al. 2024).

### 4.2 Oxidative Stress

Probiotic supplementation resulted in significantly greater improvements between groups in antioxidant defenses and oxidative stress markers (*p* < 0.001 for all: increased TAC and GPx; decreased TOS and MDA), with the probiotic group showing favorable changes (e.g., TAC +0.40 vs. −0.01 mmol/L; MDA −0.046 vs. +0.006 nmol/mL) while the placebo group exhibited worsening or minimal change in several markers despite energy surplus. This may reflect paradoxical oxidative stress from increased substrate load or incomplete metabolic adaptation in undernourished states, consistent with reports in vulnerable populations. These findings were corroborated by studies in patients with diabetic foot ulcers, where probiotics reduced MDA and increased TAC (Mohseni et al. 2018), and in metabolic syndrome, where GPx and TOS were improved by probiotic administration (Zolghadrpour et al. 2024). A meta‐analysis reported a significant reduction in MDA and increase in TAC at probiotic doses below 0.4 × 10^10^ CFU (Musazadeh et al. 2023). Inconsistent results in other studies may stem from variations in probiotic strains or the severity of oxidative stress in different populations (Den et al. 2020; Vaghef‐Mehrabany et al. 2016). Probiotics likely exert antioxidant effects by modulating gut microbiota to produce SCFAs, chelating pro‐oxidant metal ions (e.g., iron, copper), and upregulating enzymes such as GPx and superoxide dismutase, thereby reducing reactive oxygen species and lipid peroxidation (Averina et al. 2021; Mounir et al. 2022; Xu et al. 2021). These effects are particularly relevant in undernutrition, where nutrient deficiencies exacerbate oxidative damage (Berger 2005).

### 4.3 Inflammation

The probiotic group exhibited significantly greater reductions in inflammatory markers compared with placebo (*p* < 0.001 for CRP: −0.50 vs. +0.10 mg/L; ESR: −1 vs. +1 mm/h), whereas NLR and MLR showed no significant change. The modest increases in CRP and ESR observed in the placebo group indicate that energy surplus alone is insufficient to mitigate inflammation when gut‐mediated mechanisms remain unaddressed.

Although the observed biomarker alterations achieved statistical significance, their clinical implications in undernourished adults merit consideration. Decreases in CRP and ESR, coupled with enhanced TAC, reflect attenuation of low‐grade systemic inflammation and oxidative stress, potentially diminishing infection susceptibility and facilitating immune reconstitution (Merker et al. 2020). These findings are consistent with established probiotic‐mediated anti‐inflammatory mechanisms, including modulation of gut microbiota composition, diminution of lipopolysaccharide (LPS) translocation through reinforced intestinal barrier integrity, and modulation of cytokine profiles (e.g., reduced IL‐6, elevated IL‐10) (Chen et al. 2024; Cristofori et al. 2021). SCFAs, notably butyrate, further inhibit pro‐inflammatory signaling pathways such as NF‐κB. The limited magnitude of CRP reduction and the absence of changes in NLR/MLR likely stem from the low baseline inflammatory burden and the relatively short intervention period. Collectively, these findings suggest that the tested multi‐strain probiotics confer targeted anti‐inflammatory effects in undernutrition, particularly in contexts of compromised gut barrier function (Cristofori et al. 2021).

The results of our study suggest that multi‐strain probiotic supplementation combined with an energy‐surplus diet effectively reduced gut permeability, oxidative stress, and inflammation in undernourished adults. To the best of our knowledge, this is the first RCT evaluating this specific Lactobacillus combination in underweight adults. However, the 8‐week duration limits conclusions on long‐term effects, which should be explored in future studies.

### 4.4 Conclusion and Future Directions

In conclusion, adding this multi‐strain probiotic to a 500 kcal/day energy‐surplus diet significantly improved gut barrier function, reduced oxidative stress, and attenuated inflammation in undernourished adults compared to diet alone. These findings suggest that energy surplus by diet alone may be insufficient for resolving oxidative/inflammatory issues in undernutrition, supporting adjunctive probiotics. Future research should examine longer interventions, microbiota profiling, clinical outcomes, and feasible delivery methods in diverse populations. This study employed a per‐protocol analysis to assess the efficacy of the administered dose of probiotics under optimal compliance conditions. While the low dropout rate suggests that intention‐to‐treat (ITT) findings would likely be similar, the per‐protocol approach was chosen to directly address the study's primary objective of evaluating the biological effect of the intervention when fully administered. However, this approach limits the generalizability of findings to real‐world settings where adherence may vary. The findings our study reported what could be expected if full compliance was achieved; therefore, further studies should evaluate the effects of the multi‐strain probiotic supplement on the outcomes among undernourished adults based on intention‐to‐treat analysis.

## Author Contributions

**Alireza Hatami:** conceptualization, writing – original draft, writing – review and editing, data curation, investigation, project administration, supervision, visualization, methodology. **Mohammad Hadi Eskandari:** resources, writing – review and editing. **Ali Jafarzadeh Esfehani:** formal analysis, writing – review and editing, software, visualization. **Asma Afshari:** supervision, writing – review and editing, methodology, resources. **Maryam Ahmadi‐Khorram:** conceptualization, writing – original draft, writing – review and editing, data curation, investigation, project administration, supervision, methodology. **Mohsen Nematy:** supervision, funding acquisition, resources, validation, conceptualization, project administration. **Parastoo Asghari:** writing – original draft, writing – review and editing. **Elyas Nattagh‐Eshtivani:** writing – review and editing, formal analysis.

## Funding

This study was financially supported by a grant from Mashhad University of Medical Sciences (MUMS), Mashhad, Iran (4012306).

## Ethics Statement

This study was conducted according to the principles of the Declaration of Helsinki. The protocol of this study was confirmed by the ethics committee of Mashhad University of Medical Sciences (ethics numbers: IR.MUMS.MEDICAL.REC.1401.589, IR.MUMS.MEDICAL.REC.1402.057). Written informed consent was obtained from all participants prior to enrollment after full explanation of the study purpose, procedures, potential risks and benefits, voluntary nature, right to withdraw at any time, and data confidentiality measures.

## Consent

The authors have nothing to report.

## Conflicts of Interest

The authors declare no conflicts of interest.

## Data Availability

The data that support the findings of this study are available from the corresponding author upon reasonable request.
